# Associations Between Climate‐Sensitive Nutrients, Clinical Malaria, and Anaemia Among Young Children in Rural Burkina Faso: An Analysis of Baseline Data From a Cluster‐Randomised Controlled Trial

**DOI:** 10.1111/tmi.70056

**Published:** 2025-10-16

**Authors:** Adi Lukas Kurniawan, Maria Virginia Borga, Fanta Zerbo, Carol Akinyi Abidha, Anais Gonnet, Raissa Sorgho, Boubacar Coulibaly, Ali Sié, Ina Danquah

**Affiliations:** ^1^ Transdisciplinary Research Area (TRA) “Technology and Innovation for Sustainable Futures” and Center for Development Research (ZEF), Rheinische Friedrich‐Wilhelms University of Bonn Bonn Germany; ^2^ Heidelberg Institute of Global Health (HIGH), Medical Faculty and University Hospital, Heidelberg University Heidelberg Germany; ^3^ Universidad Católica de Santa Fe Santa FE Argentina; ^4^ Centre de Recherche en Santé de Nouna Nouna Burkina Faso; ^5^ Public Health Institute (PHI), Center for Wellness and Nutrition (CWN) California USA

**Keywords:** anaemia, Burkina Faso, malaria, nutrient patterns, young children

## Abstract

**Objective:**

Malaria and anaemia are significant public health challenges among young children in Burkina Faso, with complex interactions between climate variability, food security, and nutrient deficiencies. This study aimed to identify the malariometric profile and nutrient patterns among young children and to determine the associations of nutrient patterns with clinical malaria and anaemia.

**Methods:**

This cross‐sectional study recruited 701 children aged 6–23 months in Nouna, Burkina Faso. Dietary intakes were collected using a semi‐quantitative Food Propensity Questionnaire. Principal Component Analysis was used to derive nutrient patterns. We calculated logistic regressions for the associations of nutrient patterns with clinical malaria (*Plasmodium* species with fever (≥ 37.5°C) or a history of fever within the last 2 weeks or prescribed anti‐malaria medication) and anaemia (Hb < 11 g/dL).

**Results:**

In this study population (*n* = 452; median age: 17 months (interquartile range: 12, 21); male sex: 52%), 25.0% of the children had clinical malaria, and 88.5% had anaemia. Adherence to a ‘fat and vitamin A’ dietary pattern was inversely associated with the chance of clinical malaria (OR quintile 3 vs. quintile 1: 0.50, 95% CI: 0.26, 0.96) but not with anaemia. A ‘fibre and micronutrient’ pattern was neither associated with malaria nor with anaemia.

**Conclusion:**

Anaemia is common in this malaria‐endemic area, and diets characterised by fat and vitamin A may reduce the risk of clinical malaria in young children. This highlights the need for comprehensive management strategies addressing both malaria and anaemia, including community‐based educational interventions to enhance complementary feeding practices in malaria‐endemic regions.

## 1 Introduction

Malaria and anaemia constitute significant public health challenges among children aged < 5 years in sub‐Saharan Africa [1, 2]. In Burkina Faso, malaria transmission is high and largely seasonal, and malaria remains a leading cause of morbidity and mortality among children under 5 years of age [3]. The occurrence of clinical malaria in rural Burkina Faso is notably high, with studies reporting incidence rates of up to 1.03 episodes per child in the central‐west areas and 1.18 episodes per child in the south‐west areas during the first year of life [1, 4]. In the Saponé health district, the cumulative incidence of malaria was 67.4% in a passive detection cohort and 86.2% in an active detection cohort among children under 5 years of age [5]. Another prevalent condition among children in Burkina Faso is anaemia. It is defined as a low haemoglobin concentration (Hb) and results from inadequate dietary intake and frequent episodes of infectious diseases. A recent study has revealed staggering statistics, with 90.9% of children aged 6 to 59 months in the Eastern region of Burkina Faso being anaemic [6]. Furthermore, the interaction between malaria and anaemia creates a vicious cycle; children with anaemia are more susceptible to severe malaria, which in turn exacerbates their Hb status [7, 8].

In Burkina Faso, these health issues are aggravated by complex socio‐economic and environmental factors, including climate variability [9]. Malaria is known to be highly sensitive to climatic factors such as temperature, rainfall, and humidity, which directly impact the breeding and survival of malaria vectors [10]. The interplay between climate variability and food security further complicates this situation. Climate change can lead to reduced crop yields and plant nutrient concentrations, such as iron, zinc, selenium, vitamin A, and protein—termed ‘climate‐sensitive nutrients’, thereby increasing the risk of infection, undernutrition, and subsequent anaemia among children [11, 12]. However, the existing studies on the association between clinical malaria and climate‐sensitive nutrients are inconclusive [13]. For vitamin A, a study has shown that vitamin A supplements may have a protective effect on morbidity and mortality associated with malaria. On the other hand, zinc is important for the immune system and child growth; however, its effect on malaria remains uncertain [13, 14]. Lastly, although iron deficiency partially protects against clinical malaria, iron supplementation appears to have deleterious effects in malaria‐endemic areas [15, 16].

Therefore, this study aimed to identify the associations of climate‐sensitive nutrients with clinical malaria and anaemia among young children living in rural Burkina Faso. The specific objectives were to determine the children's malariometric profile, identify nutrient patterns, and examine the associations of the identified nutrient patterns with clinical malaria and anaemia.

## 2 Methods

### 2.1 Study Design and Recruitment

This study used baseline cross‐sectional data from a cluster‐randomised control trial on children aged 6–23 months living in the Nouna area of Northwestern Burkina Faso [17]. The baseline survey was conducted from July to September 2021. In brief, we recruited 701 children at the age of complementary feeding (6–23 months) within the framework of the Nouna Health and Demographic Surveillance System (HDSS). This HDSS was established in the early 1990s and includes 59 neighbouring villages, with 115,000 inhabitants in 14,000 households, covering a total area of 1775 km^2^.

The eligibility criteria for participating households were: being a permanent resident within the HDSS area, residing within a 10‐km radius of one out of five local weather stations, having at least one child at the age of 6 to 23 months, having access to at least 40 square metres of land and a water source for irrigation, and providing informed written consent from the caregiver. The procedures for this study were approved by the Ethical Committee of Heidelberg University (S‐91/2019) and the Ethical Committee of Centre de Recherche en Santé de Nouna (CERS/2020‐6‐098).

### 2.2 Data Collection

Anthropometric assessments, including weight and height, were conducted twice on children while wearing light clothing, to one decimal point using a calibrated weighing scale (SECA 874, Germany) and an infantometre (SECA 417, Germany). Recumbent length was measured for children below 85 cm in height who were unable to stand. The weight‐for‐height z‐score (WHZ), weight‐for‐age z‐score (WAZ), and height‐for‐age or length‐for‐age z‐score (HAZ) were calculated according to the WHO reference values to determine undernutrition status, including wasting (WHZ < −2), underweight (WAZ < −2), and stunting (HAZ < −2).

Finger‐prick blood samples were collected, and Hb was measured using the HemoCue Haemoglobin 201+ analyser (HemoCue, Radiometer, Denmark). Anaemia was defined as Hb < 11 g/dL. The presence of *Plasmodium* parasites was assessed through microscopic examination of 3% Giemsa‐stained thick (45 min, pH 7.2) and thin blood film (Olympus CX22 binocular microscope, 100 × 100). To determine parasitaemia, we counted the number of asexual parasites against 200 leucocytes, and parasite density was calculated assuming 8000 leukocytes/μL blood. The body temperature (T) was measured using a digital thermometer, with current fever defined as T ≥ 37.5°C. Caregivers were asked about the participating children's history of fever within the last 2 weeks, and any use of anti‐malarial medication was documented. Clinical malaria was defined as microscopically detected *Plasmodium* parasites plus current fever or history of fever or currently prescribed anti‐malaria medication. Malarial anaemia was defined as the presence of *Plasmodium* parasites and Hb < 11 g/dL.

The children's habitual dietary intake over 6 months was assessed by a culturally adapted semi‐quantitative Food Propensity Questionnaire (FPQ) with 134 food items [18]. These food items were collapsed into 29 food groups based on their culinary use and nutrient profiles. The FPQ estimated the intake frequencies in predefined portion sizes, which were then translated into energy and nutrient intakes using the West African Food Composition database (FAO, 2012).

### 2.3 Data Analysis

The data analysis was performed using STATA version 16 (STATA Corp LLC, Texas, USA). Participants with missing data on any of the dietary variables and malaria‐related variables were excluded from the analysis. Initially, we screened 701 children, and 249 were excluded from the analysis due to missing *Plasmodium* test and missing FPQ data, resulting in a final sample of 452 children. We did mean imputation on the missing value of the mother's age (*n* = 204).

For the distributions of malaria‐related variables by gender and for the sociodemographic, anthropometric, and dietary variables by malaria status, the data are displayed as medians and interquartile ranges (IQR) for continuous variables and numbers (proportions) for categorical variables. Chi‐square tests and Mann–Whitney tests were performed to assess the significance of between‐group differences for categorical and continuous variables, respectively.

To identify nutrient patterns, we performed Principal Component Analysis (PCA) with varimax rotation to ensure that the identified pattern scores remained uncorrelated. The patterns were extracted based on the eigenvalue (> 1), the scree plot, and the interpretability of the patterns (i.e., more than 3 nutrients with factor loading ≥ 0.3). Each participant was assigned a pattern score, calculated by summing up the standardized nutrient intakes multiplied by their respective factor loading. The distributions of anthropometric, nutrient, and malaria‐related characteristics were calculated across quartiles of pattern adherence.

In logistic regression models, we calculated odds ratios (ORs), 95% confidence intervals (CIs), and *p*‐values for the associations of nutrient patterns with clinical malaria and anaemia. We calculated the associations across quartiles (with the first quartile as reference) and per 1 score‐SD increase. We fitted three models: the crude model without adjustment; Model 1 with adjustments for the strongest risk factors of undernutrition and malaria: age, gender, breastfeeding status, and total energy intake; and Model 2 with additional adjustments for socio‐economic covariables: caregiver's education, marital status, ethnicity, occupation, household size, and number of children under five within the household. A *p*‐value of < 0.05 was considered statistically significant.

## 3 Results

Among the 452 children examined, 43 (9.5%) had a microscopically visible infection with *Plasmodium* parasites, all of which were *P. falciparum*. Current fever was observed in 62 children (13.7%), while a history of fever was documented for 180 children (39.8%). Considering the number of children with *P. falciparum* infection, being on anti‐malarial medication, having fever or a history of fever, 113 (25.0%) children were defined as having clinical malaria. The median Hb was 9.4 (IQR: 8.4, 10.2) g/dL. Anaemia was identified in 400 children (88.5%) (boys: 210; 88.6%), and 37 children (8.2%) had malarial anaemia (Table 1). Median Hb was significantly different between boys and girls (boys: 9.3 vs. girls: 9.6 g/dL, *p* = 0.038). All other characteristics did not show significant differences in their distribution between boys and girls.

**TABLE 1:** Malariometric characteristics of children living in the Nouna HDSS area.

Characteristics	Total	Boys	Girls	*p*
*N*	452	237	215	
Age (months)	17 (12, 21)	18 (13, 21)	16 (11, 20)	0.021
Hb (g/dL)	9.4 (8.4, 10.2)	9.3 (8.2, 10.1)	9.6 (8.5, 10.4)	0.038
Anaemia (< 11 g/dL)	400 (88.5)	210 (88.6)	190 (88.4)	0.938
Fever (≥ 37.5°C)	62 (13.7)	35 (14.8)	27 (12.6)	0.495
History of fever (yes)	180 (39.8)	100 (42.2)	80 (37.2)	0.280
History of malaria (yes)	66 (14.6)	40 (16.9)	26 (12.1)	0.150
Prescribed malaria medication (yes)	101 (45.3)	56 (44.8)	45 (45.9)	0.868
*P. falciparum* infection (yes)	43 (9.5)	24 (10.1)	19 (8.8)	0.641
Clinical malaria (yes)	113 (25.0)	62 (26.2)	51 (23.7)	0.550
Malarial anaemia (yes)	37 (8.2)	20 (8.4)	17 (7.9)	0.837

*Note:* Data are presented as medians (interquartile ranges) for continuous variables and as numbers (percentages) for categorical variables. *p*‐values were calculated using the Mann–Whitney U‐test for continuous variables and the chi‐square test for categorical variables. *P. falciparum* infection: based on expert microscopy. Clinical malaria: *P. falciparum* infection plus current fever or history of fever or prescribed anti‐malaria medication. Malarial anaemia: *P. falciparum* infection plus Hb < 11 g/dL.

Table 2 shows the socio‐demographic, anthropometric, and dietary characteristics of children stratified by their clinical malaria status. No statistically significant differences were observed between children with and without clinical malaria for the median anthropometric z‐scores and for the consumption of energy and nutrients. Similarly, there were no differences in the socio‐demographic characteristics between these groups.

**TABLE 2:** Sociodemographic, anthropometric, and dietary characteristics of children living in the Nouna HDSS area, by clinical malaria status.

Characteristics	Total	No malaria	Malaria	*p*
*N*	452	339	113	
*Sociodemographic*				
Age (months)	17 (12, 21)	17 (12, 21)	17 (13, 21)	0.621
Gender				0.550
Boys	237 (52.4)	175 (51.6)	62 (54.9)	
Girls	215 (47.6)	164 (48.4)	51 (45.1)	
Age of the mother (years)	32 (30, 32)	32 (31, 32)	32 (26, 32)	0.128
Mother's education				0.812
None	323 (73.6)	241 (72.8)	82 (75.9)	
Primary	42 (9.6)	34 (10.3)	6 (7.4)	
Secondary	55 (12.5)	41 (12.4)	14 (13.0)	
Post‐secondary	19 (4.3)	15 (4.5)	4 (3.7)	
Mother's primary occupation, subsistence farming	403 (91.8)	306 (92.4)	97 (89.8)	0.819
Mother's marital status				0.565
Single/widowed/divorced	150 (33.8)	116 (34.6)	34 (31.2)	
Married/cohabiting	292 (65.8)	217 (64.8)	75 (68.8)	
Don't know	2 (0.4)	2 (0.6)	0 (0.0)	
Major ethnic group, Dafing	228 (51.3)	171 (51.0)	57 (52.3)	0.821
Number of people in the household	12 (8, 18)	12 (8, 19)	11 (8, 18)	0.321
Number of children under 5 years in the household	2 (2, 4)	2 (2, 4)	2 (2, 3)	0.672
Breastfeeding, yes	395 (87.4)	297 (87.6)	98 (86.7)	0.806
*Anthropometric*				
Weight‐for‐age z‐score (WAZ)	−0.99 (−1.96, −0.18)	−0.96 (−2.0, −0.06)	−1.07 (−1.88, −0.38)	0.411
Weight‐for‐height z‐score (WHZ)	−0.66 (−1.67, 0.35)	−0.65 (−1.71, 0.41)	−0.75 (−1.53, 0.19)	0.918
Height‐for‐age z‐score (HAZ)	−1.08 (−2.06, −0.02)	−1.01 (−2.04, 0.01)	−1.1 (−2.09, −0.31)	0.382
Underweight (WAZ < −2)	104 (24.4)	80 (25.2)	24 (22.2)	0.540
Wasting (WHZ < −2)	77 (18.1)	64 (20.1)	13 (12.0)	0.059
Stunting (HAZ < −2)	117 (27.3)	86 (26.9)	31 (28.7)	0.712
*Dietary*				
Energy intake (kcal/day)	1156.4 (588.8, 1889.1)	1133.4 (563.7, 1877.7)	1235.1 (631.0, 1975.5)	0.501
Carbohydrates (energy %)	57.8 (49.6, 64.6)	57.1 (49.6, 64.3)	58.8 (49.2, 65.4)	0.336
Protein (energy %)	13.0 (11.4, 15.1)	13.1 (11.4, 15.2)	12.9 (11.3, 14.5)	0.449
Total fat (energy %)	27.8 (22.3, 36.2)	28.0 (22.7, 36.1)	26.5 (21.3, 36.3)	0.286
Fibre (g/day)	20.0 (9.5, 32.0)	19.1 (9.3, 31.6)	22.8 (10.1, 35.1)	0.236
Retinol equivalents (μg/day)	545.7 (237.3, 1307.2)	543.7 (237.7, 1131.9)	551.0 (236.1, 1607.8)	0.708
Selenium (μg/day)	54.1 (28.9, 112.8)	53.4 (28.5, 112.5)	58.8 (31.4, 114.9)	0.524
Zinc (mg/day)	6.7 (4.0, 11.9)	6.5 (4.0, 11.9)	7.5 (4.5, 11.9)	0.687
Iron (mg/day)	9.0 (4.7, 14.7)	8.9 (4.7, 14.4)	9.5 (4.7, 14.9)	0.453

*Note:* Data are presented as medians (interquartile ranges) for continuous variables and as numbers (percentages) for categorical variables. *p*‐values were analysed using Mann–Whitney U‐test for continuous variables and chi‐square test for categorical variables.

**FIGURE 1:**
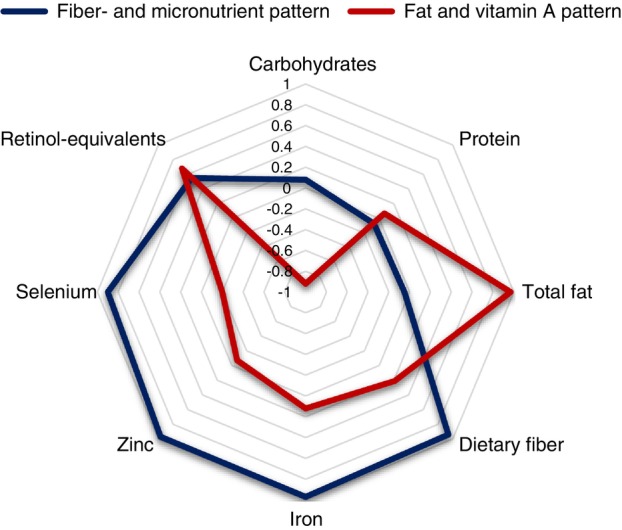
Rotated factor loadings of two exploratory nutrient patterns among children living in the Nouna HDSS area.

Figure 1 shows two identified nutrient patterns and their rotated factor loadings. A ‘fibre‐ and micronutrient’ pattern was characterised by positive factor loadings of dietary iron, zinc, selenium, fibre, and retinol equivalents. It explained 48.9% of the variation in nutrient intakes. The ‘fat and vitamin A’ pattern showed positive factor loadings of total fat and retinol‐equivalents and a negative correlation with carbohydrate intake. This pattern explained 29.6% of the variation in nutrient intakes. The malariometric, anthropometric, and dietary characteristics of children across quartiles of the nutrient patterns are presented in Table S1. There were positive trends in nutrient intakes across increasing pattern quartiles(*p* < 0.001) for both patterns, except for carbohydrate in the ‘fibre‐ and micronutrient’ pattern and for selenium in the ‘fat and vitamin A’ pattern. Notably, in Q3 of the ‘fat and vitamin A’ pattern, which has a median of retinol equivalent 707.7 μg/day, we observed the fewest cases of clinical malaria (18.6%) and malarial anaemia (2.7%).

Tables 3 and 4 present the associations between nutrient patterns and clinical malaria and anaemia, respectively. In Model 3, there were non‐significant associations between quartiles of the ‘fibre‐and micronutrient’ pattern and clinical malaria. Likewise, a 1‐SD increase in the ‘fibre‐ and micronutrient’ pattern score was not associated with clinical malaria. However, the ‘fat and vitamin A’ pattern showed a significant inverse association with clinical malaria (Q3 OR: 0.50, 95% CI: 0.26–0.96, *p* = 0.036) in Model 3 (Table 3). Yet, this association was non‐linear with the lowest number of cases in quartile 3. Table 4 shows that neither the ‘fibre‐ and micronutrient’ pattern nor the ‘fat and vitamin A' pattern was associated with anaemia.

**TABLE 3:** Associations of nutrient patterns with clinical malaria among 452 children living in the Nouna HDSS area.

Nutrient pattern	Clinical malaria (*n*)	Odds ratio (95% confidence intervals) for clinical malaria					
		Crude	*p*	Model 1	*p*	Model 2	*p*
Fibre‐ and micronutrient pattern							
Quartile 1	28/113	Reference					
Quartile 2	26/113	0.91 (0.49, 1.67)	0.755	0.83 (0.43, 1.63)	0.595	0.96 (0.48, 1.91)	0.907
Quartile 3	29/113	1.05 (0.57, 1.91)	0.878	0.90 (0.41, 1.98)	0.798	1.10 (0.48, 2.51)	0.825
Quartile 4	30/113	1.10 (0.60, 1.99)	0.761	0.84 (0.26, 2.75)	0.779	1.03 (0.30, 3.53)	0.968
Per 1 score‐SD increase		1.02 (0.97, 1.08)	0.441	1.17 (0.83, 1.64)	0.370	1.23 (0.86, 1.75)	0.248
Fat and vitamin A pattern							
Quartile 1	33/113	Reference					
Quartile 2	27/113	0.76 (0.42, 1.38)	0.367	0.71 (0.39, 1.30)	0.266	0.70 (0.37, 1.31)	0.259
Quartile 3	21/113	0.55 (0.30, 1.03)	0.063	0.52 (0.27, 0.98)	**0.043**	0.50 (0.26, 0.96)	**0.036**
Quartile 4	32/113	0.96 (0.54, 1.70)	0.883	0.90 (0.50, 1.63)	0.736	1.05 (0.55, 1.98)	0.889
Per 1 score‐SD increase		0.98 (0.89, 1.08)	0.731	0.98 (0.89, 1.08)	0.614	0.99 (0.89, 1.10)	0.872

*Note:* Model 1: adjusted for children's age, gender, breastfeeding status, and total energy. Model 2: additionally adjusted for mother's age, ethnicity, mother's education, marital status, occupation, household size, and number of children under five. Bold values indicate statistical significance (*p* < 0.05).

**TABLE 4:** Associations of nutrient patterns with anaemia (Hb < 11 g/dL) among 452 children living in the Nouna HDSS area.

Nutrient pattern	anaemia (*n*)	Odds ratio (95% confidence intervals) for anaemia					
		Crude	*p*	Model 1	*p*	Model 2	*p*
Fibre‐ and micronutrient pattern							
Quartile 1	106/113	Reference					
Quartile 2	97/113	0.40 (0.16, 1.01)	0.054	0.39 (0.14, 1.05)	0.063	0.40 (0.14, 1.10)	0.077
Quartile 3	102/113	0.61 (0.23, 1.64)	0.330	0.60 (0.18, 2.01)	0.410	0.73 (0.21, 2.49)	0.613
Quartile 4	95/113	0.35 (0.14, 0.87)	**0.024**	0.31 (0.06, 1.57)	0.157	0.38 (0.07, 1.96)	0.248
Per 1 score‐SD increase		0.94 (0.88, 1.01)	0.089	0.72 (0.46, 1.13)	0.149	0.71 (0.45, 1.13)	0.152
Fat and vitamin A pattern							
Quartile 1	101/113	Reference					
Quartile 2	102/113	1.10 (0.46, 2.61)	0.826	1.30 (0.54, 3.14)	0.556	1.80 (0.71, 4.54)	0.213
Quartile 3	95/113	0.63 (0.29, 1.37)	0.242	0.73 (0.33, 1.63)	0.442	0.97 (0.41, 2.25)	0.935
Quartile 4	102/113	1.10 (0.46, 2.61)	0.826	1.30 (0.53, 3.15)	0.567	1.33 (0.51, 3.46)	0.559
Per 1 score‐SD increase		0.97 (0.86, 1.10)	0.641	0.99 (0.87, 1.13)	0.885	0.98 (0.84, 1.13)	0.757

*Note:* Model 1: adjusted for children's age, gender, breastfeeding status, and total energy. Model 2: additionally adjusted for mother's age, ethnicity, mother's education, marital status, occupation, household size, and number of children under five. Bold values indicate statistical significance (*p* < 0.05).

## 4 Discussion

In this study, we examined the associations of climate‐sensitive nutrient patterns with clinical malaria and anaemia among 452 young children in rural Burkina Faso. This cross‐sectional analysis revealed that 25.0% of the children had clinical malaria, and anaemia was present in 88.5%. Two nutrient patterns were identified using PCA: the ‘fibre and micronutrient’ pattern (accounting for a 48.9% variation in nutrient intakes) and the ‘fat and vitamin A’ pattern (accounting for a 29.6% variation in nutrient intakes). Higher adherence to the ‘fat and vitamin A’ pattern was associated with a reduced chance of clinical malaria (OR Q3 vs. Q1: 0.50, 95% CI: 0.26, 0.96). This association was non‐linear and plateaued in quartile 3. No association was seen with anaemia. Also, for the ‘fibre and micronutrient’ pattern, no statistically significant associations were observed, neither with clinical malaria nor with anaemia.

In our study, the proportion of children with anaemia (88.5%) accords with previously reported numbers. Anaemia prevalence among children aged 6 to 59 months in Burkina Faso is reported to be exceptionally high, with figures ranging from 74% to 88%, and in specific rural areas, it can reach 97%, with more than 20% experiencing severe forms of the condition [19, 20]. This prevalence is significantly above the global estimate of 40% for this age group, according to the World Health Organization (WHO) [21]. In previous studies in Burkina Faso, a higher prevalence of anaemia was observed in regions affected by food insecurity and poor agricultural conditions, which have been mainly attributed to adverse weather conditions [22]. This high prevalence also seems attributable to a combination of nutritional deficiencies and the impact of infectious diseases such as malaria. The role of malaria in exacerbating anaemia is particularly pronounced in Burkina Faso, where the disease is endemic. Among children under 5 years of age, the prevalence of *Plasmodium* spp. infection is estimated at 38%, with district‐specific proportions ranging from 11% to 78% [23]. Clinical malaria cases were documented at 47% among children during their first year of life in the central‐west region of Burkina Faso [1]. Our findings are consistent with these figures on the prevalence of *Plasmodium* spp. infection in the Nouna region (10%). Clinical malaria significantly reduces Hb in children due to haemolysis of erythrocytes, increased elimination of infected erythrocytes, and inflammation‐related impairment of erythropoiesis [24]. A recent study that pooled individual‐level nationally representative HDSS data indicates that acute malaria can decrease population‐level Hb by as much as 7.7 g/dL (95% CI: −8.8, −6.6) [19]. The rainy season, which typically spans from June to November and coincides with our recruitment period, usually exhibits a marked increase in malaria cases, leading to higher incidences of anaemia during this period [5]. Nevertheless, malarial anaemia was relatively uncommon in our study population, as indicated by the low prevalence of malarial anaemia cases (8.2%) compared to the high overall prevalence of anaemia. Therefore, the observed associations between nutrient patterns and haemoglobin levels are more likely to reflect dietary causes of anaemia rather than the direct effects of *Plasmodium* spp. infection, thus indicating that other factors, such as nutritional deficiencies, play a more dominant role in this context. The variation in age range of children and implementation of seasonal malaria chemoprevention (SMC) may contribute to the low prevalence of *Plasmodium* spp. infection and the number of clinical cases. Notably, the nationwide rollout of SMC since 2014 has achieved high coverage, with up to 100% of targeted children receiving preventive treatment during the high transmission season [3]. This implementation of SMC has been a critical strategy aiming at reducing malaria incidence among young children, yet the effectiveness of this intervention varies, while the burden of malaria shifts towards older children as younger cohorts receive preventive treatments [3, 25]. Collectively, this underscores the need for comprehensive management strategies that address both clinical malaria and anaemia among young children in Burkina Faso.

Concerning dietary intake, children in our study primarily consumed carbohydrate‐based food (58% of energy), while the intakes of total fat (28% of energy) and protein (13% of energy) were lower than the respective dietary recommendations [26]. Indeed, starchy staples, such as millet‐based dishes, constitute the predominant sources of energy in the children's diets in rural Burkina Faso. These foods are accompanied by dried okra and red sorrel leaf vegetables, providing calories but lacking essential protein, fat, and micronutrients—necessary for optimal growth and development [27]. For the climate‐sensitive micronutrients vitamin A, selenium, zinc, and iron, our findings suggest that dietary intakes might have been adequate in this population, as evidenced by median values falling within the Estimated Average Requirements (EARs) [28]. However, this interpretation should be considered with caution, as the dietary assessment tool has not been calibrated to the portion sizes of the target age group, which may result in overestimation. The identified nutrient patterns, derived from the calculated nutrient intakes in the FPQ data, reflect the profiles of nutrients commonly consumed together. The ‘fibre‐ and micronutrient’ pattern may indicate plant‐based feeding practices, while the ‘fat and vitamin A’ pattern suggests the frequent consumption of yellow‐ and orange‐fleshed fruits and vegetables and animal‐based foods, such as red meat and eggs.

Our study found that none of the identified dietary patterns showed a significant association with anaemia, suggesting that micronutrient intakes are sufficient. A non‐linear inverse association was observed between higher adherence to the ‘fat and vitamin A' pattern (Q3 vs. Q1) and clinical malaria. Vitamin A has a narrow optimal intake range: deficiency and excess can lead to health problems. The median retinol‐equivalent intake for Q3 of the ‘fat and vitamin A' pattern was 707.7 μg/day, which falls within the recommended daily allowance for children of this age group [29]. This suggests that, on average, children in this quartile are likely consuming sufficient and beneficial amounts of vitamin A, thereby supporting its potential protective role against clinical malaria. Such an association was not evident for the ‘fibre and micronutrient’ pattern. Previous studies may help interpret these findings. With regard to iron, the relationship between iron status and clinical malaria is complex and multifaceted, with evidence indicating that both iron deficiency and iron excess can influence the risk of clinical malaria. Studies in East Africa have reported that iron‐deficient children experience fewer malaria episodes, which may confer protection against severe manifestations of malaria [16]. Conversely, elevated iron levels have been linked to a higher risk of malaria during the first year of life [15, 30]. Indeed, iron is an essential nutrient for the proliferation and replication of *Plasmodium* parasites. Consequently, iron supplementation must be implemented with caution, especially in highly malaria‐endemic regions. However, if appropriate antimalarial treatment and insecticide‐treated bed nets were provided, such as in Ghana, daily use of iron supplementation did not increase the incidence of clinical malaria among children aged 6 to 35 months [31]. Concerning vitamin A, numerous studies have reported the potential protective effects of vitamin A supplementation against clinical malaria [32, 33]. Additionally, randomised controlled trials in Burkina Faso and Ghana have demonstrated that combined vitamin A and zinc supplementation may exert a synergistic effect, significantly reducing malaria morbidity and resulting in 30% fewer malaria cases [34, 35]. Conversely, studies on vitamin A deficiency and with individuals who have not been supplemented with vitamin A have demonstrated increased susceptibility to *P. falciparum* infection and malaria episodes [36, 37, 38]. These findings indicate that vitamin A plays a crucial role in enhancing immune function, which may contribute to the mitigation of *Plasmodium* infections. Regarding zinc alone, this essential trace element plays a critical role in immune function, erythropoiesis, and the formation of globin chains in the haemoglobin molecule. However, as reported in several systematic reviews, its role in *Plasmodium* spp. infection and malaria morbidity remains mixed [39, 40]. Notably, these results suggest that while zinc plays a role in immune functioning, its direct effect on malaria morbidity is not straightforward [41].

The strength of this study was the inclusion of a relatively large sample size of young children living in rural Burkina Faso, which enhanced the generalizability of the findings in this region. However, the following limitations must be acknowledged. Firstly, the cross‐sectional nature of the study is susceptible to recall bias of feeding practices by caregivers, potentially leading to over‐ or underreporting of dietary intakes. Secondly, the cross‐sectional design is also prone to reverse causation. Therefore, it remains unclear whether high adherence to the ‘fat and vitamin A’ pattern has been influenced by malaria status or vice versa. Future studies—ideally prospective designs—should employ objective measurements, such as biomarkers from both invasive and non‐invasive methods, including zinc concentration, body iron storage (as measured by ferritin), and iron transport (as facilitated by transferrin). The cross‐sectional design, which captures data at a single point in time, also precludes the ability to account for seasonal variation in dietary intake and malaria incidence, which are known to fluctuate over different seasons in this setting. Thirdly, the use of the semi‐quantitative FPQ precludes the estimation of absolute food and nutrient intakes. Nevertheless, as all participants are subject to the same measurement error, this tool enabled us to rank the study participants according to their intakes. Fourthly, the transferability of our findings may be limited to regions of Burkina Faso with malaria endemicity patterns similar to the Nouna HDSS area, characterised by seasonal, high‐intensity transmission. Therefore, caution is warranted when applying these results to regions with markedly different malaria transmission dynamics. Lastly, although our study has adjusted for important demographic, clinical, and socioeconomic confounding variables, the possibility of unmeasured or residual confounding cannot be excluded.

While acknowledging the limitations, our study highlights the importance of education and community engagement in promoting nutrition knowledge and practices among caregivers of young children in malaria‐endemic areas of SSA. Raising awareness about the relationships between nutrient intakes and clinical malaria can empower communities to adopt preventive measures. Moreover, community‐based educational interventions by community health workers (CHWs), tailored to improve complementary feeding practices and introduce local foods that do not inhibit the absorption of climate‐sensitive nutrients, are beneficial [42]. Importantly, effective public health strategies for promoting healthy nutrition and controlling malaria in young children should be multifaceted, incorporating integrated interventions, community education, and targeted nutritional support. By addressing the interconnectedness of these health issues, public health initiatives can significantly enhance health outcomes in malaria‐endemic regions.

## 5 Conclusion

In conclusion, this study underlines the high prevalence of clinical malaria and anaemia among young children in rural Burkina Faso, highlighting them as persistent public health challenges in this region. While the identified dietary patterns were not associated with the chance of anaemia, higher adherence to the ‘fat and vitamin A’ pattern was inversely associated with clinical malaria, suggesting potential protective effects within the optimal ranges of fat and vitamin A intakes. These findings emphasise the necessity for integrated public health interventions that address both malaria and anaemia concurrently. Future research should adopt longitudinal designs and incorporate biomarker‐based assessments to elucidate further the role of nutrient patterns in mitigating malaria and anaemia risks.

## Ethics Statement

This study was conducted according to the Declaration of Helsinki guidelines, and all procedures involving research study participants were approved by the Ethical Committee of Heidelberg University (S‐291/2019) and the Ethical Committee of Centre de Recherche en Santé de Nouna (CERS/2020‐6‐098). Written informed consent was obtained from all caregivers.

## Conflicts of Interest

The authors declare no conflicts of interest.

## Supporting information


**Table S1:** Malariometric, anthropometric, and nutrient profiles across quartiles of nutrient patterns.

## References

[1] H. M. Natama , E. Rovira‐Vallbona , M. A. Somé , et al., “Malaria Incidence and Prevalence During the First Year of Life in Nanoro, Burkina Faso: A Birth‐Cohort Study,” Malaria Journal 17 (2018): 163, 10.1186/s12936-018-2315-4. 29650007 PMC5898041

[2] A. Björkman , “Malaria Associated Anaemia, Drug Resistance and Antimalarial Combination Therapy,” International Journal for Parasitology 32 (2002): 1637–1643, 10.1016/s0020-7519(02)00192-3. 12435448

[3] T. Druetz , N. Corneau‐Tremblay , T. Millogo , et al., “Impact Evaluation of Seasonal Malaria Chemoprevention Under Routine Program Implementation: A Quasi‐Experimental Study in Burkina Faso,” American Journal of Tropical Medicine and Hygiene 98 (2018): 524–533, 10.4269/ajtmh.17-0599. 29260654 PMC5929206

[4] A. B. Tiono , D. T. Kangoye , A. M. Rehman , et al., “Malaria Incidence in Children in South‐West Burkina Faso: Comparison of Active and Passive Case Detection Methods,” PLoS One 9 (2014): e86936, 10.1371/journal.pone.0086936. 24475198 PMC3901722

[5] A. Ouédraogo , A. B. Tiono , A. Diarra , et al., “Malaria Morbidity in High and Seasonal Malaria Transmission Area of Burkina Faso,” PLoS One 8 (2013): e50036, 10.1371/journal.pone.0050036. 23320064 PMC3540059

[6] O. Ouédraogo , E. W. R. Compaoré , O. Ouédraogo , M. Kiburente , and M. H. Dicko , “Prevalence and Associated Factors of Anemia in Children Aged 6 to 59 Months in the Eastern Region of Burkina Faso,” Global Pediatric Health 11 (2024): 2333794X241263163, 10.1177/2333794X241263163. PMC11268009 39049881

[7] S. Diallo , S. A. Roberts , S. Gies , et al., “Malaria Early in the First Pregnancy: Potential Impact of Iron Status,” Clinical Nutrition 39 (2020): 204–214, 10.1016/j.clnu.2019.01.016. 30737046 PMC6660428

[8] A. Deribew , F. Alemseged , F. Tessema , et al., “Malaria and Under‐Nutrition: A Community Based Study Among Under‐Five Children at Risk of Malaria, South‐West Ethiopia,” PLoS One 5 (2010): e10775, 10.1371/journal.pone.0010775. 20505829 PMC2874013

[9] C. Caminade , K. M. McIntyre , and A. E. Jones , “Impact of Recent and Future Climate Change on Vector‐Borne Diseases,” Annals of the New York Academy of Sciences 1436 (2019): 157–173, 10.1111/nyas.13950. 30120891 PMC6378404

[10] Q. Liu , Y. Wang , J. Deng , et al., “Association of Temperature and Precipitation With Malaria Incidence in 57 Countries and Territories From 2000 to 2019: A Worldwide Observational Study,” Journal of Global Health 14 (2024): 04021, 10.7189/jogh.14.04021. 38385445 PMC10882640

[11] S. S. Myers , M. R. Smith , S. Guth , et al., “Climate Change and Global Food Systems: Potential Impacts on Food Security and Undernutrition,” Annual Review of Public Health 38 (2017): 259–277, 10.1146/annurev-publhealth-031816-044356. 28125383

[12] M. R. Smith and S. S. Myers , “Impact of Anthropogenic CO_2_ Emissions on Global Human Nutrition,” Nature Climate Change 8 (2018): 834–839, 10.1038/s41558-018-0253-3.

[13] R. M. Peñuela , L. V. Agudelo , E. M. Tamayo , et al., “Malaria and Malnutrition in Children and Household Food Insecurity: A Review,” Perspectives in Public Health 11 (2009): 55–70.

[14] S. Basnet , M. Mathisen , and T. A. Strand , “Oral Zinc and Common Childhood Infections—An Update,” Journal of Trace Elements in Medicine and Biology 31 (2015): 163–166, 10.1016/j.jtemb.2014.05.006. 24906347

[15] V. Moya‐Alvarez , G. Cottrell , S. Ouédraogo , M. Accrombessi , A. Massougbodgi , and M. Cot , “High Iron Levels Are Associated With Increased Malaria Risk in Infants During the First Year of Life in Benin,” American Journal of Tropical Medicine and Hygiene 97 (2017): 497–503, 10.4269/ajtmh.16-0001. 28722565 PMC5544062

[16] C. K. Bundi , A. Nalwoga , L. Lubyayi , et al., “Iron Deficiency Is Associated With Reduced Levels of Plasmodium Falciparum‐Specific Antibodies in African Children,” Clinical Infectious Diseases 73 (2021): 43–49, 10.1093/cid/ciaa728. 32507899 PMC8246895

[17] I. Mank , R. Sorgho , F. Zerbo , et al., “ALIMUS‐We Are Feeding! Study Protocol of a Multi‐Center, Cluster‐Randomized Controlled Trial on the Effects of a Home Garden and Nutrition Counseling Intervention to Reduce Child Undernutrition in Rural Burkina Faso and Kenya,” Trials 23 (2022): 449, 10.1186/s13063-022-06423-5. 35650583 PMC9157031

[18] C. Galbete , M. Nicolaou , K. A. Meeks , et al., “Food Consumption, Nutrient Intake, and Dietary Patterns in Ghanaian Migrants in Europe and Their Compatriots in Ghana,” Food & Nutrition Research 61, no. 1 (2017): 1341809, 10.1080/16546628.2017.1341809. 28747862 PMC5510194

[19] T. Starck , C. A. Bulstra , H. Tinto , et al., “The Effect of Malaria on Haemoglobin Concentrations: a Nationally Representative Household Fixed‐Effects Study of 17,599 Children Under 5 Years of Age in Burkina Faso,” Malaria Journal 20 (2021): 416, 10.1186/s12936-021-03948-z. 34688294 PMC8542337

[20] A. N. Zeba , H. Z. Ouedraogo , and A. Hien , “Impacts of Malaria on Severe Anemia in Children Aged 6‐23 Months Old From the Rural District of Kongoussi, Burkina Faso,” Journal of Nutrition and Food Security 7 (2022): 496–507.

[21] R. J. Soares Magalhães and A. C. Clements , “Spatial Heterogeneity of Haemoglobin Concentration in Preschool‐Age Children in Sub‐Saharan Africa,” Bulletin of the World Health Organization 89 (2011): 459–468, 10.2471/BLT.10.083568. 21673862 PMC3099553

[22] Y. C. Chuang , T. W. Chuang , H. J. Chao , et al., “Contextual Factors and Spatial Patterns of Childhood Malnutrition in Provinces of Burkina Faso,” Journal of Tropical Pediatrics 66 (2020): 66–74, 10.1093/tropej/fmz031. 31086979

[23] M. Ouédraogo , S. Samadoulougou , T. Rouamba , et al., “Spatial Distribution and Determinants of Asymptomatic Malaria Risk Among Children Under 5 Years in 24 Districts in Burkina Faso,” Malaria Journal 17 (2018): 460, 10.1186/s12936-018-2606-9. 30526598 PMC6286519

[24] N. J. White , “Anaemia and malaria,” Malaria Journal 17 (2018): 371, 10.1186/s12936-018-2509-9. 30340592 PMC6194647

[25] J. B. Yaro , A. B. Tiono , A. Ouedraogo , et al., “Risk of Plasmodium Falciparum Infection in South‐West Burkina Faso: Potential Impact of Expanding Eligibility for Seasonal Malaria Chemoprevention,” Scientific Reports 12 (2022): 1402, 10.1038/s41598-022-05056-7. 35082312 PMC8791962

[26] A. Sié , C. Tapsoba , C. Dah , et al., “Dietary Diversity and Nutritional Status Among Children in Rural Burkina Faso,” International Health 10 (2018): 157–162, 10.1093/inthealth/ihy016. 29579204 PMC5915942

[27] J. E. Arsenault , L. Nikiema , P. Allemand , et al., “Seasonal Differences in Food and Nutrient Intakes Among Young Children and Their Mothers in Rural Burkina Faso,” Journal of Nutritional Science 3 (2014): e55, 10.1017/jns.2014.53. 26101623 PMC4473133

[28] Y. Martin‐Prevel , P. Allemand , L. Nikiema , et al., “Biological Status and Dietary Intakes of Iron, Zinc and Vitamin A Among Women and Preschool Children in Rural Burkina Faso,” PLoS One 11, no. 3 (2016): e0146810, 10.1371/journal.pone.0146810. 26991908 PMC4798773

[29] S. Awasthi and A. Awasthi , “Role of Vitamin A in Child Health and Nutrition,” Clinical Epidemiology and Global Health 8 (2020): 1039–1042, 10.1016/j.cegh.2020.03.016.

[30] J. M. Muriuki , A. J. Mentzer , W. Kimita , et al., “Iron Status and Associated Malaria Risk Among African Children,” Clinical Infectious Diseases 68 (2019): 1807–1814, 10.1093/cid/ciy791. 30219845 PMC6522755

[31] S. Zlotkin , S. Newton , A. M. Aimone , et al., “Effect of Iron Fortification on Malaria Incidence in Infants and Young Children in Ghana: a Randomized Trial,” Journal of the American Medical Association 310 (2013): 938–947, 10.1001/jama.2013.277129. 24002280

[32] F. Sandalinas , S. Filteau , E. J. M. Joy , L. Segovia de la Revilla , A. MacDougall , and H. Hopkins , “Measuring the Impact of Malaria Infection on Indicators of Iron and Vitamin A Status: A Systematic Literature Review and Meta‐Analysis,” British Journal of Nutrition 129 (2023): 87–103, 10.1017/S0007114522000757. 35260210 PMC9816655

[33] M. A. Sanjoaquin and M. E. Molyneux , “Malaria and Vitamin A Deficiency in African Children: a Vicious Circle?,” Malaria Journal 8 (2009): 134, 10.1186/1475-2875-8-134. 19534807 PMC2702350

[34] A. N. Zeba , H. Sorgho , N. Rouamba , et al., “Major Reduction of Malaria Morbidity With Combined Vitamin A and Zinc Supplementation in Young Children in Burkina Faso: A Randomized Double Blind Trial,” Nutrition Journal 7 (2008): 7, 10.1186/1475-2891-7-7. 18237394 PMC2254644

[35] S. Owusu‐Agyei , S. Newton , E. Mahama , et al., “Impact of Vitamin A With Zinc Supplementation on Malaria Morbidity in Ghana,” Nutrition Journal 12 (2013): 131, 10.1186/1475-2891-12-131. 24330422 PMC3850154

[36] V. Nankabirwa , T. Tylleskar , J. Nankunda , et al., “Malaria Parasitaemia Among Infants and Its Association With Breastfeeding Peer Counselling and Vitamin A Supplementation: A Secondary Analysis of a Cluster Randomized Trial,” PLoS One 6 (2011): e21862, 10.1371/journal.pone.0021862. 21760916 PMC3131393

[37] O. A. Lawal , S. A. Adegoke , S. B. Oseni , and O. A. Oyelami , “Low Serum Vitamin A Is Prevalent in Underfive Children With Severe Malaria and Is Associated With Increased Risk of Death,” Journal of Infection in Developing Countries 12 (2018): 365–372, 10.3855/jidc.9572. 31865300

[38] A. H. Shankar , B. Genton , R. D. Semba , et al., “Effect of Vitamin A Supplementation on Morbidity due to Plasmodium Falciparum in Young Children in Papua New Guinea: a Randomised Trial,” Lancet 354 (1999): 203–209, 10.1016/S0140-6736(98)08293-2. 10421302

[39] M. Kotepui , P. Wilairatana , W. Mala , K. U. Kotepui , F. R. Masangkay , and K. Wangdi , “Effects of Daily Zinc Alone or in Combination With Other Nutrient Supplements on the Risk of Malaria Parasitaemia: A Systematic Review and Meta‐Analysis of Randomised Controlled Trials,” Nutrients 15 (2023): 2855, 10.3390/nu15132855. 37447182 PMC10346149

[40] M. Y. Yakoob , E. Theodoratou , A. Jabeen , et al., “Preventive Zinc Supplementation in Developing Countries: Impact on Mortality and Morbidity due to Diarrhea, Pneumonia and Malaria,” BMC Public Health 11, no. Suppl 3 (2011): S23, 10.1186/1471-2458-11-S3-S23. 21501441 PMC3231897

[41] J. Veenemans , P. Milligan , A. M. Prentice , et al., “Effect of Supplementation With Zinc and Other Micronutrients on Malaria in Tanzanian Children: A Randomised Trial,” PLoS Medicine 8 (2011): e1001125, 10.1371/journal.pmed.1001125. 22131908 PMC3222646

[42] V. Owino , C. Kumwenda , B. Ekesa , et al., “The Impact of Climate Change on Food Systems, Diet Quality, Nutrition, and Health Outcomes: A Narrative Review,” Frontiers in Climate 4 (2022): 941842, 10.3389/fclim.2022.941842.

