# Supplementary material for: Associations Between Climate‐Sensitive Nutrients, Clinical Malaria, and Anaemia Among Young Children in Rural Burkina Faso: An Analysis of Baseline Data From a Cluster‐Randomised Controlled Trial
Source: Trop Med Int Health. 2025 Oct 16;31(2):181–9. doi: 10.1111/tmi.70056 (PMC12868394; doi:10.1111/tmi.70056)
Supplement: Supplementary file 1 — Table S1: Malariometric, anthropometric, and nutrient profiles across quartiles of nutrient patterns. [file TMI-31-181-s001.docx]

Supplementary Table 1. Malariometric, anthropometric, and nutrient profiles across quartiles of nutrient patterns

|  | **Q1 (n = 113)** | **Q2 (n = 113)** | **Q3 (n = 113)** | **Q4 (n = 113)** | **P** |
| --- | --- | --- | --- | --- | --- |
| **Fibre- and micronutrient pattern** | | | | | |
| Gender |  |  |  |  | 0.131 |
| Boys | 52 (46.0) | 65 (57.5) | 66 (58.4) | 54 (47.8) |  |
| Girls | 61 (54.0) | 48 (42.5) | 47 (41.6) | 59 (52.2) |  |
| Age (months) | 15 (10, 21) | 17 (11, 21) | 17 (14, 21) | 18 (14, 21) | 0.001 |
| **Malariometric** |  |  |  |  |  |
| Hb (g/dL) | 9.4 (8.3, 10.1) | 9.5 (8.4, 10.4) | 8.9 (7.9, 9.9) | 9.7 (9.0, 10.6) | 0.026 |
| Anaemia (<11g(dL) | 106 (93.8) | 97 (85.8) | 102 (90.3) | 95 (84.1) | 0.092 |
| Fever (≥37.5°C) | 18 (15.9) | 16 (14.2) | 17 (15.0) | 11 (9.7) | 0.538 |
| History of fever (yes) | 41 (36.3) | 42 (37.2) | 52 (46.0) | 45 (39.8) | 0.435 |
| History of malaria (yes) | 15 (13.3) | 11 (9.7) | 19 (16.8) | 21 (18.6) | 0.242 |
| Prescribed malaria medication (yes) | 24 (47.1) | 21 (38.9) | 27 (40.3) | 29 (56.9) | 0.223 |
| Malaria infection (yes) | 11 (9.7) | 16 (14.2) | 9 (8.0) | 7 (6.2) | 0.204 |
| Clinical malaria (yes) | 28 (24.8) | 26 (23.0) | 29 (25.7) | 30 (26.6) | 0.938 |
| Malarial anaemia (yes) | 9 (8.0) | 15 (13.3) | 8 (7.1) | 5 (4.4) | 0.102 |
| **Anthropometric** |  |  |  |  |  |
| Weight-for-age Z score (WAZ) | -0.8 (-1.9, -0.1) | -0.9 (-1.9, 0.1) | -1.1 (-2.2, -0.5) | -1.2 (-1.8, -0.4) | 0.410 |
| Weight-for-height Z score (WHZ) | -0.7 (-1.7, 0.4) | -0.5 (-1.4, 0.8) | -0.7 (-1.7, 0.0) | -0.9 (-1.6, 0.1) | 0.381 |
| Height-for-age Z score (HAZ) | -1.0 (-2.0, 0.2) | -1.3 (-2.3, -0.1) | -1.0 (-2.3, -0.0) | -1.0 (-1.9, -0.2) | 0.995 |
| Underweight (WAZ >-2) | 24 (22.9) | 25 (23.2) | 34 (31.2) | 21 (20.2) | 0.267 |
| Wasting (WHZ >-2) | 25 (23.4) | 35 (32.4) | 33 (30.3) | 24 (23.1) | 0.299 |
| Stunting (HAZ >-2) | 21 (20.0) | 18 (16.7) | 22 (20.2) | 16 (15.4) | 0.743 |
| **Nutrients** |  |  |  |  |  |
| Energy intake (kcal/day) | 264.7 (120.4, 389.7) | 867.3 (746.7, 1049.9) | 1465.6 (1251.5, 1686.8) | 2503.4 (2137.1, 2887.4) | <0.001 |
| Carbohydrates (energy %) | 26.7 (45.3, 66.8) | 61.5 (56.3, 67.5) | 53.6 (37.6, 62.0) | 57.1 (51.3, 62.4) | 0.413 |
| Protein (energy %) | 14.0 (12.1, 16.6) | 13.0 (11.4, 15.4) | 12.4 (10.3, 14.2) | 13.1 (11.6, 14.5) | <0.001 |
| Total fat (energy %) | 28.0 (20.0, 37.0) | 24.8 (21.0, 29.4) | 31.2 (24.6, 52.3) | 29.5 (23.7, 36.0) | 0.008 |
| Fibre (g/day) | 3.0 (1.0, 6.3) | 13.8 (10.8, 17.4) | 27.8 (23.3, 29.5) | 40.4 (35.4, 50.9) | <0.001 |
| Retinol-equ (µg/day) | 105.9 (29.5, 171.5) | 372.0 (263.6, 510.7) | 1055.2 (653.4, 3548.7) | 1541. 2 (852.6, 3939.9) | <0.001 |
| Selenium (µg/day) | 13.0 (5.3, 21.9) | 45.9 (38.8, 58.8) | 75.6 (42.5, 113.4) | 148.5 (105.0, 188.4) | <0.001 |
| Zinc (mg/day) | 1.6 (0.7, 2.6) | 5.9 (5.3, 6.7) | 9.1 (5.9, 10.9) | 14.7 (13.1, 17.1) | <0.001 |
| Iron (mg/day) | 1.6 (0.7, 2.8) | 7.0 (5.3, 8.1) | 11.3 (9.6, 13.1) | 19.6 (17.1, 22.7) | <0.001 |
| **Fat and vitamin A pattern** | | | | | |
| Gender |  |  |  |  | 0.745 |
| Boys | 56 (49.6) | 64 (56.6) | 59 (52.2) | 58 (51.3) |  |
| Girls | 57 (50.4) | 49 (43.4) | 54 (47.8) | 55 (48.7) |  |
| Age (months) | 15 (10, 21) | 19 (14, 22) | 17 (14, 21) | 16 (12, 20) | 0.275 |
| **Malariometric** |  |  |  |  |  |
| Hb (g/dL) | 9.0 (8.0, 10.0) | 9.5 (8.4, 10.3) | 9.6 (8.6, 10.4) | 9.5 (8.4, 10.2) | 0.030 |
| Anaemia (<11g(dL) | 101 (89.4) | 102 (90.3) | 95 (84.1) | 102 (90.3) | 0.399 |
| Fever (≥37.5°C) | 13 (11.5) | 17 (15.0) | 13 (11.5) | 19 (16.8) | 0.569 |
| History of fever (yes) | 40 (35.4) | 51 (45.1) | 39 (34.5) | 50 (44.3) | 0.212 |
| History of malaria (yes) | 15 (13.3) | 16 (14.2) | 16 (14.2) | 19 (16.8) | 0.888 |
| Prescribed malaria medication (yes) | 29 (51.8) | 21 (37.5) | 19 (36.5) | 32 (54.2) | 0.120 |
| Malaria infection (yes) | 15 (13.3) | 13 (11.5) | 5 (4.4) | 10 (8.9) | 0.120 |
| Clinical malaria (yes) | 33 (29.2) | 27 (23.9) | 21 (18.6) | 32 (28.3) | 0.232 |
| Malarial anaemia (yes) | 13 (11.5) | 13 (11.5) | 3 (2.7) | 8 (7.1) | 0.044 |
| **Anthropometric** |  |  |  |  |  |
| Weight-for-age Z score (WAZ) | -1.2 (-2.0, -0.4) | -0.9 (-2.0, 0.1) | -0.9 (-1.7, -0.2) | -1.0 (-2.3, -0.2) | 0.410 |
| Weight-for-height Z score (WHZ) | -0.7 (-1.7, -0.1) | -0.2 (-1.4, 0.8) | -0.7 (-1.6, 0.2) | -0.9 (-0.22, 0.4) | 0.350 |
| Height-for-age Z score (HAZ) | -1.0 (-1.9, -0.2) | -1.3 (-2.6, -0.2) | -0.9 (-1.9, 0.1) | -1.1 (-2.0, -0.0) | 0.901 |
| Underweight (WAZ >-2) | 28 (25.0) | 25 (23.4) | 18 (17.0) | 33 (32.7) | 0.072 |
| Wasting (WHZ >-2) | 21 (18.8) | 14 (13.1) | 16 (15.1) | 26 (25.7) | 0.090 |
| Stunting (HAZ >-2) | 26 (23.0) | 39 (36.5) | 26 (24.5) | 26 (25.5) | 0.105 |
| **Nutrients** |  |  |  |  |  |
| Energy intake (kcal/day) | 842.8 (385.5, 1449.1) | 115.2 (750.4, 1808.1) | 1182.0 (460.4, 1989.1) | 1338.7 (1097.2, 2291.8) | <0.001 |
| Carbohydrates (energy %) | 69.6 (66.8, 73.1) | 61.2 (58.7, 62.8) | 53.2 (50.3, 55.3) | 41.3 (36.6, 47.2) | <0.001 |
| Protein (energy %) | 11.7 (10.3, 12.9) | 13.9 (12.7, 15.1) | 14.9 (13.4, 16.6) | 11.9 (10.2, 14.2) | <0.001 |
| Total fat (energy %) | 18.8 (15.7, 20.9) | 25.0 (23.7, 26.6) | 31.4 (29.9, 34.2) | 43.7 (38.5, 52.9) | <0.001 |
| Fibre (g/day) | 14.5 (6.3, 23.5) | 19.2 (11.0, 30.0) | 16.6 (6.5, 33.5) | 29.3 (23.6, 41.3) | <0.001 |
| Retinol-equ (µg/day) | 262.6 (143.0, 471.9) | 543.7 (285.7, 816.6) | 707.7 (293.4, 1184.2) | 3560.9 (1198.6, 4105.5) | <0.001 |
| Selenium (µg/day) | 47.9 (22.8, 112.8) | 61.5 (37.6, 129.5) | 66.1 (28.2, 129.2) | 46.6 (30.5, 96.8) | 0.842 |
| Zinc (mg/day) | 5.7 (2.6, 8.7) | 7.6 (5.3, 12.6) | 7.8 (3.5, 12.4) | 6.1 (4.5, 12.4) | 0.033 |
| Iron (mg/day) | 7.2 (2.7, 10.3) | 9.0 (5.4, 13.6) | 8.8 (3.0, 15.9) | 10.4 (8.6, 17.9) | <0.001 |
| Data are presented as medians (interquartile ranges) for continuous variables and as numbers (percentages) for categorical variables. P-values were analysed using the GLM for continuous variables and the chi-square test for categorical variables | | | | | |
